# Correction: Urea and water are transported through different pathways in the red blood cell membrane

**DOI:** 10.1085/jgp.20221332208082023c

**Published:** 2023-08-22

**Authors:** Jesper Brahm, Morten Hanefeld Dziegiel, Jonas Leifelt

Vol. 155, No. 8 | https://doi.org/10.1085/jgp.202213322 | June 30, 2023

We regret that, during production of the article, the half-time of the efflux process in Equation 3 was mistakenly changed. The corrected equation appears below. The error appears in PDFs downloaded before August 10, 2023.

$$P=k\times \frac{V_{i}}{A}=\frac{\ln2}{T_{1/2}}\times \frac{V_{i}}{A}$$

